# p.L105Vfs mutation in a family with thymic neuroendocrine tumor combined with MEN1: a case report

**DOI:** 10.1186/s12883-020-01659-7

**Published:** 2020-03-04

**Authors:** Hongjuan Zheng, Shishi Zhou, Wanfen Tang, Qinghua Wang, Xia Zhang, Xiayun Jin, Ying Yuan, Jianfei Fu

**Affiliations:** 1 Department of Medical Oncology, Jinhua Hospital, Zhejiang University School of Medicine, 351 Mingyue Road, Jinhua, 321000 Zhejiang Province China; 2 Department of Medical Oncology, Second Affiliated Hospital of Zhejiang University School of Medicine, Hangzhou, 310000 China

**Keywords:** Thymic neuroendocrine tumors, MEN1, Mutation

## Abstract

**Background:**

Multiple endocrine neoplasia type 1 (MEN1) is a rare autosomal dominant disorder arising from mutations of the MEN1 tumor suppressor gene on chromosome 11q13; MEN1 is characterized by the development of neuroendocrine tumors, including those of the parathyroid, gastrointestinal endocrine tissue and anterior pituitary. Additionally, thymic neuroendocrine tumors in MEN1 are also rarely reported.

**Case presentation:**

This case report observed a family that presented with MEN1 p.L105Vfs mutation, and two of the family members had been diagnosed with thymic neuroendocrine tumor combined with MEN1. To the best of our knowledge, this is the first time such a mutation in the MEN1 gene has been reported. The proband presented with thymic neuroendocrine tumor, parathyroid adenoma and rectum adenocarcinoma. The son of the proband presented with thymic neuroendocrine tumor, gastrinoma, hypophysoma and parathyroid adenoma. Genetic testing revealed the frameshift mutation p.L105Vfs, leading to the identification of one carrier in the pedigree (the patient’s younger sister). The proband then underwent parathyroidectomy at the age of 26 years (in 1980) for a parathyroid adenoma. Subsequently, the patient underwent thymectomy, radiotherapy and chemotherapy. The patient is now 64 years old, still alive and still undergoing Lanreotide therapy.

**Conclusion:**

Thymic neuroendocrine MEN1 is rare, but it accounts for almost 20% of MEN1-associated mortality. Consequently, we should focus on regular clinical screening of the thymus in MEN1 patients.

## Background

Thymic tumors are a relatively rare type of solid tumor in the chest, and the incidence of thymic tumors is approximately 1–10/100,000 [1–3]. Additionally, thymic neuroendocrine tumors (th-NETs) are rarer than thymic tumors, representing approximately 2–5% of all thymic tumors, and only 25% of th-NETs are also diagnosed with MEN1 [4]. Th-NETs are classified using the newest 4th edition of the World Health Organization (WHO) criteria into low-grade typical carcinoids, intermediate-grade atypical carcinoids, and two high-grade malignancies, large cell neuroendocrine carcinoma and small cell carcinoma [5, 6]. Besides, mitotic count and Ki-67 index have been used to grade th-NETs into NET G1, NET G2, NET G3 and thymic neuroendocrine carcinoma according to a molecular classification of th-NET [7].

Multiple endocrine neoplasia type 1 (MEN1) is an infrequent disease with an incidence of approximately 2~20/100,000 [8–10] that is characterized by tumors of the parathyroid, pancreas, or anterior pituitary. Other tumors, such as gastrinoma, carcinoid, adrenal cortical tumors, angiofibroma, collagenoma, lipoma, and thymoma, also occur in some patients. Additionally, there are 3–8% of MEN1 patients involved with th-NET. However, some studies found that th-NET accounted for about 20% of the total deaths in patients with MEN1 [11, 12]. Thus, there is an important clinical significance to investigate MEN1 in families with th-NET.

## Case presentation

**Fig. 1:**
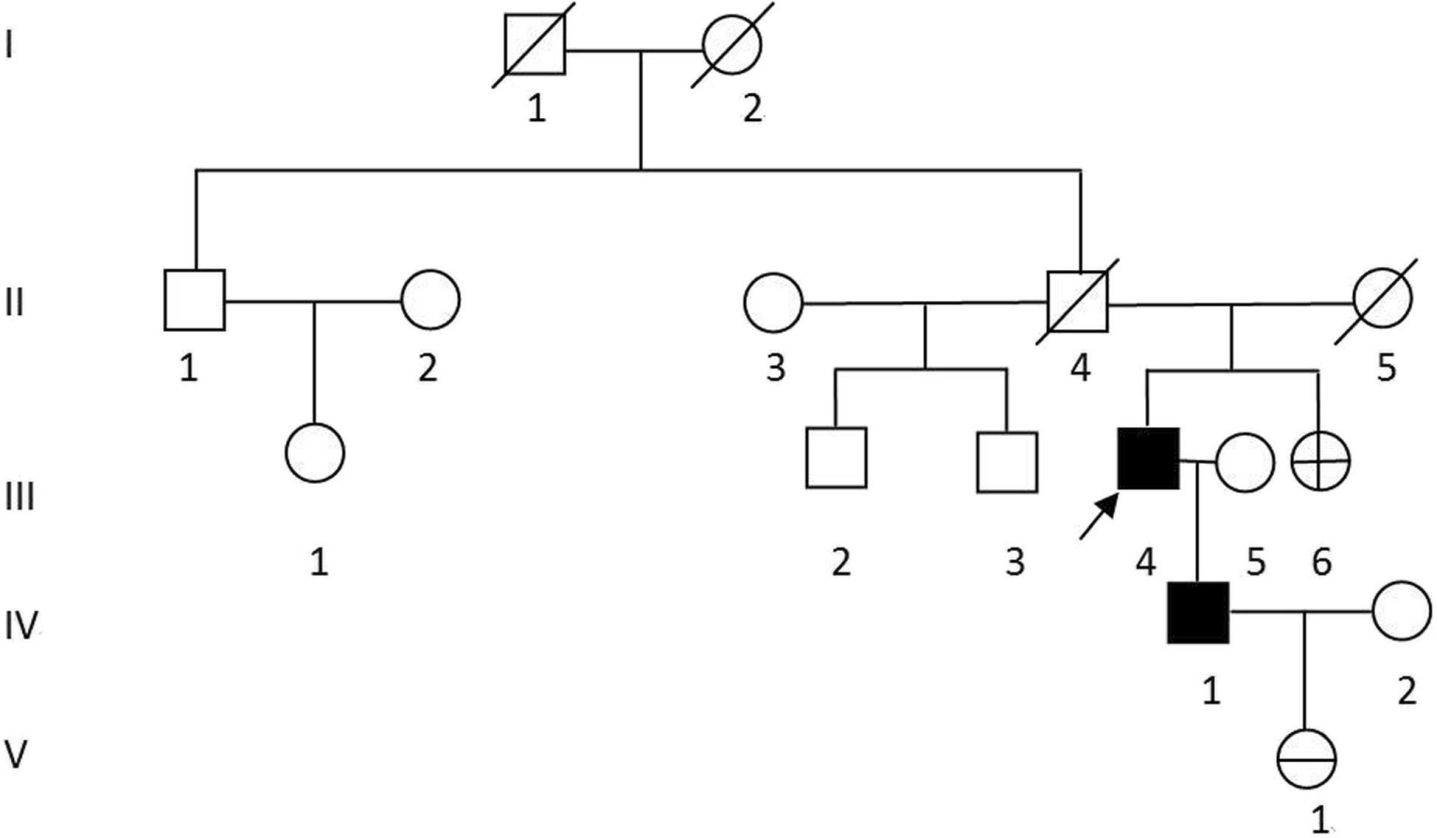
Pedigree of a family with MEN1. Legend: Generations available for study are indicated by Roman numerals I, II, III, IV and V. Black symbol, affected subjects; square, male; circle, female. / indicates a deceased individual at the time of the investigation, and the arrow indicates the presence of a proband. + in circle, MEN1 mutation carrier; − in circle, negative in MEN1 mutation gene test

**Fig. 2:**
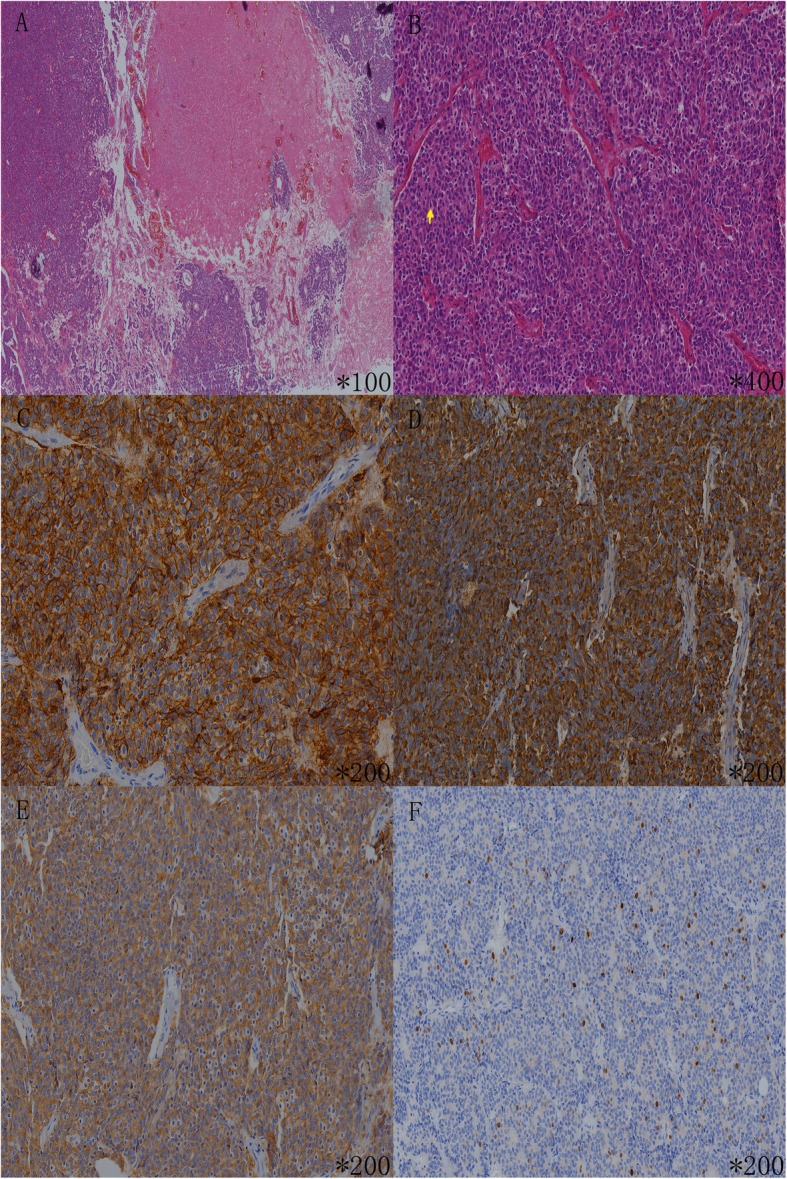
Pathological and immunohistochemical results of the proband. Legend: On hematoxylin and eosin stain, the tumor cells proliferated with a solid nest-like pattern, and were separated by slight fibrous vascular septum (**a**). The tumor cells show round to oval, hyperchromatic nuclei and the nucleoli of the tumor cells were inconspicuous, and there were a few mitotic figures (7~8/ 10HPF) (**b**). On immunohistochemical stain, the tumor cells were strong positive for CD56 (**c**), CgA (**d**) and Syn (**e**), and the Ki-67 proliferation index is estimated at 3% (**f**). The yellow arrow in B is mitotic figure

The proband in the pedigree (Fig. 1), a 64-year-old man, was admitted to a local hospital with coughing for a week. He was already diagnosed with thymic neuroendocrine tumor since the age of 52 years (in 2006). Therefore, systemic radiological examination was performed at our hospital. Enhanced computed tomography (CT) of the chest revealed an anterior superior mediastinal tumor, and magnetic resonance imaging (MRI) of the epigastrium found multiple circular cystic masses in the pancreas. The level of serum pro-gastrin-releasing peptide (ProGRP) was 214 pg/ml (reference range: 2–50 pg/ml). No other tumorous lesions were found. Thus, resection of thymic neoplasms was performed in the same year. The postoperative pathology was th-NET. Histologically, the tumor cells show round to oval, hyperchromatic nuclei, the nucleoli of the tumor cells were inconspicuous, and there were a few mitotic figures (7~8/10 HPF). On immunohistochemical staining, the tumor cells were strongly positive for synaptophysin (Syn), chromogranin (CgA) and CD56, and the Ki-67 proliferation index was estimated at 3% (Fig. 2). Above all, the pathologists diagnosed the tumor as thymic neuroendocrine tumor, WHO type atypical carcinoid. At the age of 60 years (in 2014), imaging revealed local recurrence of thymoma combined with multiple bone metastases, and then the patient underwent radiotherapy, T5/L3 kyphoplasty and chemotherapy. Until now, the patient is still undergoing Lanreotide therapy.

### Previous history

The proband was diagnosed with parathyroid adenoma in 1980 with a history of parathyroid gland surgery. Presently, color doppler ultrasonography of the thyroid showed bilateral lobectomy and nodules (size: 1.0*0.8 cm) on the dorsal side of the right lobe, diagnosed as parathyroid hyperplasia (PTU: 140 pg/ml; reference range, 15–65 pg/ml). Urological ultrasound showed multiple urinary tract calculi. He was diagnosed as moderately differentiated adenocarcinoma (T2N0M0) on March 29, 2017.

**Fig. 3:**
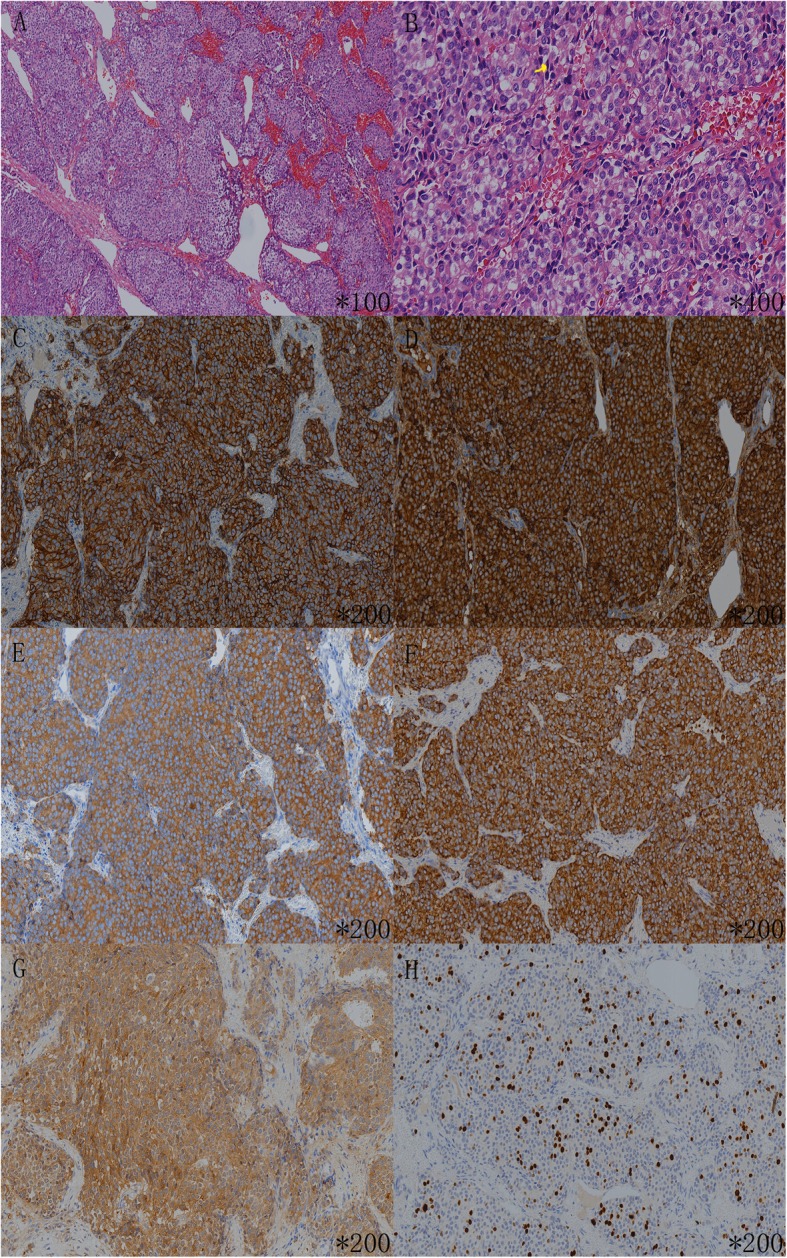
Pathological and immunohistochemical results of the IV-1. Legend: On hematoxylin and eosin stain, the tumor cells proliferate with a solid nest-like pattern, and are separate by slight fibrous vascular septum (**a**). The tumor cells show round to oval, hyperchromatic nuclei and the nucleoli of the tumor cells are inconspicuous, and there were a few mitotic figures (8~10/ 10HPF) (**b**). On immunohistochemical stain, the tumor cells were strong positive for CD56 (**c**), CgA (**d**), Syn (**e**), CK (**f**) and NSE (**g**), and the Ki-67 proliferation index is estimated at 30% (H). The yellow arrow in B is mitotic figure

The IV-1 in the pedigree (Fig. 1), 38 years old, is the proband’s son, who was admitted to the hospital with right chest pain for more than 20 days at the age of 33 years (in 2013). CT of the chest revealed a huge mass in the anterior mediastinum. The results of core needle biopsy suggested a neuroendocrine tumor of the thymic gland, and there were a few mitotic figures (8~10/10 HPF). On immunohistochemical staining, the tumor cells were strongly positive for Syn, CgA, CD56, CK-pan, epithelial membrane antigen (EMA), and neuron-specific enolase (NSE). Additionally, the Ki-67 proliferation index was estimated at 30% (Fig. 3). A pancreatic neoplasm was found by enhanced CT of the abdomen, and the level of serum gastrin was 82.3 pg/ml (reference range: 50–150 pg/ml). MRI of the brain revealed hypophysoma. Endocrine investigation revealed a serum prolactin level of 2642.69 mIU/L (reference range for males: 54.69–384.14 mIU/L). Ultrasound pictures of the thyroid showed a nodule (size, 1.0*0.6 cm) on the right parathyroid gland, and the level of parathyroid hormone was 98 pg/ml (reference range: < 70 pg/ml). Above all, IV-1 was diagnosed with th-NET, gastrinoma, hypophysoma and parathyroid adenoma. IV-1 was diagnosed with unresectable disease and thus received systemic treatment. And he was died on March 19, 2019.

### Family history

The pedigree of this family is shown in Fig. 1. Four members (III-4, III-6, IV-1 and V-1) of the family were screened. The proband’s parents are dead without a tumor history. He has an uncle who also has no history of cancer, but the daughter of his uncle (III-1) was diagnosed with lung cancer at 32 years old. The brothers and a sister of the proband are in good health without thymus, parathyroid gland, or pancreatic space-occupying disease, and their blood biochemistry indicates blood calcium levels within the normal range. IV-1, the son of the proband, was diagnosed with th-NET, gastrinoma, hypophysoma and parathyroid adenoma (see above for details).

### MEN1 gene detection results

**Fig. 4:**
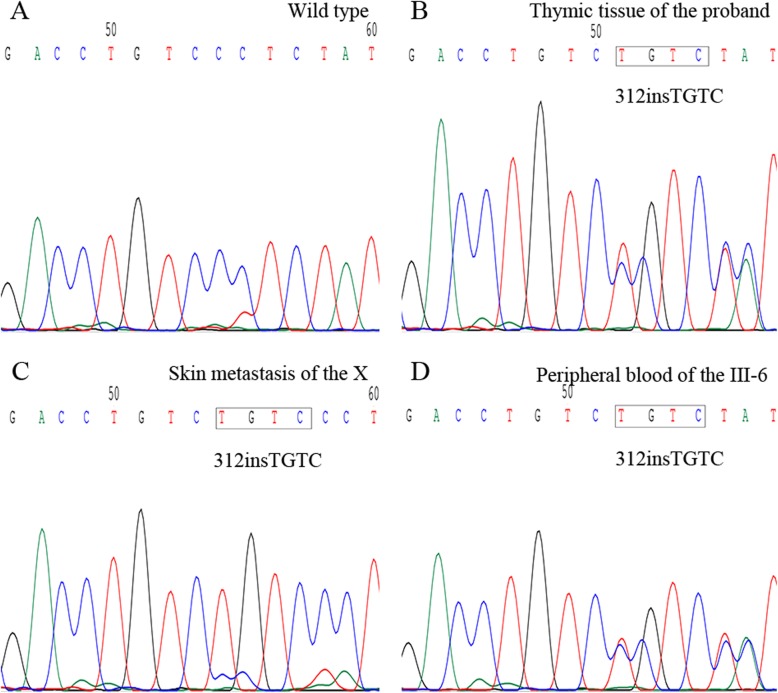
Genetic test results of the family. The insertion of 312GTGC results in a frameshift mutation of MEN1: p.L105Vfs mutation

A frameshift mutation (p.L105Vfs) of MEN1 was detected in the thymic neuroendocrine tumor of the proband. (Fig. 4-b). The frameshift mutation (p.L105Vfs) of MEN1 was detected in the skin metastatic lesion of IV-1 (Fig. 4-c). Germline heterozygous mutation of MEN1 p.L105Vfs was found in the proband, IV-1 and III-6 rather than in V-1by testing their white blood cells. Germline mutation testing was checked by next-generation sequencing.

## Discussion

According to some studies [13–15], there may be no difference in clinical features between th-NET MEN1 patients and regular MEN1 patients. In this case, both the proband and IV-1 had parathyroid lesions and th-NET. Additionally, IV-1 presented with a pituitary tumor. Therefore, the proband and his son could be definitively diagnosed with th-NET MEN1.

The age of diagnosis in 95% of patients with MEN1 developing clinical manifestations was more than 50 years old previously [13]. With the development of diagnostic techniques, more and more patients have been diagnosed with MEN1 in their fourth decades in a recent report [16]. In the current family, the proband was diagnosed with th-NET combined with MEN1 at the age of 52 years in 2006. IV-1 was diagnosed at the age of 33 years with a huge mass in the anterior mediastinum. To date, Goudet P et al. have reported the youngest case with th-NET MEN1 at the age of 16 years [14]. MEN1 is difficult to be distinguished from normal neuroendocrine tumors. Therefore, the diagnosis of MEN1 in young groups should be mainly determined from germline mutation screening. In the current family, in the absence of screening, IV-1 failed to be diagnosed earlier, resulting in missed optimal opportunities for radical surgery. Thus, we suggest that clinical screening in families with MEN1 mutation should be started earlier than 30 years old, which was also recommended in Thakker’s study [17].

Until now, in Europe and the United States, several retrospective studies [4, 14, 15, 18, 19] showed that 95–100% of patients with th-NET MEN1 were male. A Japanese study [10] reported 28 patients with th-NET MEN1 in the national database; and only 63% of these patients were male. In China, Ye reported 9 patients with th-NET MEN1, and only 4 patients were male [16]. The reason for this ethnic difference remains unclear and requires further investigation. However, in our study, both cases were male. Additionally, III-6, as a female with germline mutation of MEN1, did not show evidence of th-NET. The reason for the male predominance remains unclear. All MEN1 patients and asymptomatic gene mutation carriers, especially males, should be warned of the risk of th-NET.

MEN1 gene cloning has benefited from the two-hit hypothesis of Knudson [20]. The first hit leads to MEN1 gene heterozygous mutation of genital cells, and the second hit was certain chromosome deletion of somatic or tumor cells. The mutation of the MEN1 gene is often heterozygous mutation, and cell function can be normal; however, once the normal allelic gene develops a deletion, loss of heterozygosity (LOH) will occur, which will lead to the loss of large areas of normal chromosomes and tumor development [9, 13, 21, 22].

Anlauf and his co-authors found that 13/28 (46.4%) duodenal gastrin-producing neuroendocrine tumors and 2/5 (40%) somatostatin-secreting neuroendocrine tumors revealed LOH of 11q13 from six patients with Zollinger–Ellison syndrome and MEN1 [23, 24]. At the same time, none of the 12 precursor lesions showed LOH at the 11q13 locus. Therefore, the authors conclude that allelic deletion of the MEN1 gene might reflect a pivotal event in the development of neuroendocrine tumors of patients with MEN1. In our case report, we could further prove that LOH of the MEN1 gene is vital step in the development of th-NET of patients with MEN1. The proband, the father of IV-1, was older than IV-1 when th-NETs were diagnosed. Moreover, the prognosis of the proband was better than IV-1. The reason for the obvious difference in age and prognosis between the proband and IV-1 might be that LOH of MEN1 gene was detected in the skin metastatic lesion of IV-1, but not in the thymic neuroendocrine tumor of the proband.

More than 1336 MEN1 mutations have been reported in Lemos MC et al.’s study, and 40% of the 1336 mutations are insertion or deletion mutations with a frameshift, 25% are nonsense mutations, 20% are missense mutations, 5% are insertion or deletion mutations without a frameshift, and 10% are split-site mutations. Most (80%) of these mutations are predicted to lead to truncated forms of menin protein that disrupt the interactions of menin with other proteins. Ultimately, truncated menin protein will lose the ability to inhibit tumorigenesis [13]. One study [25], published in 2002, found that truncated mutations are concentrated in exons 2 and 10, while missense mutations are mostly concentrated in and near exon 3. These mean that there may be existing hot regions rather than hot spot mutations in the MEN1 gene, and it remains unclear whether there is a correlation between MEN1 gene mutation types and histopathological types and clinicopathological features. Menin is a multicellular expression nucleoprotein that serves as a tumor suppressor [26]. Menin is primarily a nuclear protein and directly binds to DNA through its COOH terminus that including three individual nuclear localization signals (NLS1, NLS2, and NLSa) [27, 28]. In this case, the frameshift mutation in exon 2 (p.L105Vfs) of MEN1, which was detected for the first time, is caused by the insertion of TGTC at the 105th codon, resulting in a frameshift mutation of MEN1 and a premature stop codon (TGA) in the 117th codon. Finally, menin lose its COOH terminus NLSs and abolish the ability to bind DNA and also fail to repress cell proliferation.

Some previous studies on th-NET MEN1 highlighted a poor prognosis because many patients die from complications of th-NET rather than dying from other manifestations of MEN1. Additionally, local invasion, recurrence, and distant metastasis are common with th-NET MEN1 [4, 14, 15]. MEN1 patients with th-NET showed a risk of death that was almost four times higher than that in patients without this type of lesion [29]. Teh BT et al.’s study reported that nine of ten th-NET MEN1 patients had died from th-NET [15]. And a systematic review by Ye revealed 40.0% of patients with th-NET had died from the disease with the shorter median survival time than regular MEN1patients [10]. Therefore, for MEN1 patients, some researchers recommend that prophylactic cervical thymectomy should be performed in both genders at the time of parathyroidectomy to prevent the subsequent development of a thymic carcinoid [18, 19].

As for clinical management of NTEs, the European Society for Medical Oncology (ESMO), the European Neuroendocrine Tumor Society (ENETS) and the National Comprehensive Cancer Network (NCCN) Guidelines recommend that surgical resection is the main therapy for localized disease, and also a choice for extensive disease. External radiotherapy, cytotoxic treatment and Interferon-Alpha can be used when disease progresses [30–32]. Besides, according to the ENETS and NCCN Guidelines [31, 32], treatment with Octreotide or Lanreotide, peptide receptor radionuclide therapy (PRRT) or targeted therapy (Everolimus) are recommended for advanced disease. ENETS [31] also recommend Interventional Radiology for selected NETs patients with high liver burden. In this case, the proband underwent thymic resection, chemotherapy and Lanreotide therapy, and now he is still alive. In conclusion, although th-NET MEN1 is rare, it accounts for almost 20% of MEN1-associated mortality. Consequently, we should focus on regular clinical screening of the thymus in MEN1 patients. To our knowledge, the p.L105Vfs mutation has never been reported in previous literature, and this mutation will produce a truncated menin protein. The specific function of this truncated menin protein remains to be further studied.

## Data Availability

Data sharing is not applicable to this article as no datasets were generated or analyzed during the current study.

## Abbreviations

CgA: Chromogranin
CT: Computed tomography
EMA: Epithelial membrane antigen
MEN1: Multiple endocrine neoplasia type 1
MRI: Magnetic resonance imaging
NLS: Nuclear localization signals
NSE: Nneuron-specific enolase
ProGRP: Pro-gastrin-releasing peptide
Syn: Synaptophysin
th-NETs: Thymic neuroendocrine tumors
TTP: Time to progression
WHO: World Health Organization
